# Effectiveness of emotion focused skills training for parents: study protocol for a randomized controlled trial in specialist mental health care

**DOI:** 10.1186/s12888-022-04084-x

**Published:** 2022-07-07

**Authors:** Linda Severinsen, Jan Reidar Stiegler, Helene Amundsen Nissen-Lie, Ben Shahar, Rune Zahl-Olsen

**Affiliations:** 1 Department of Child and Adolescent Mental Health, Sorlandet Hospital, Postboks 416, 4604 Kristiansand, Norway; 2 Department of Psychology, University of Oslo, Problemveien 7, 0315 Oslo, Norway; 3 Institute for Psychological Counseling, Chr. Michelsens gate 4, 5012 Bergen, Norway; 4 The Paul Baerwald School of Social Work and Social Welfare, The Hebrew University of Jerusalem, 91905 Jerusalem, Israel

**Keywords:** Emotion-focused skills training, Mental health care, Parents, Children and adolescent

## Abstract

**Background:**

Emotion-Focused Skills Training (EFST) is a newly developed manualized skill training program for parents to strengthen emotional bonds between parents and children and improve mental health outcomes in children. Results from several preliminary trials indicate that EFST can be quite effective, but more rigorous methods are needed to affirm the evidence of the program. The primary objective of this study is to compare the effectiveness of EFST to treatment as usual (TAU) in a Norwegian outpatient clinic for child and adolescent mental health. Additionally, the study will examine the basic theoretical assumption underlying EFST that increased parental emotional functioning predicts a decline in children’s mental health symptoms.

**Method:**

120 patients will be randomly assigned to either EFST or TAU. The main outcome measure is the semi-structured diagnostic interview Schedule for Affective Disorders and Schizophrenia, present and lifetime version (K-SADS-PL) scored by trained assessors administered at pretreatment and repeated after 3 months. The secondary outcome measure is the DSM-IV version of the Strength and Difficulties Questionnaire (SDQ) administered at pretreatment as well as 3, 6, and 12 months after the intervention. To examine the efficacy question, effect sizes and reliable change for each of the treatment arms will be assessed as well as symptom differences between the conditions. To examine the second aim of the study, we will examine (1) how parents relate to emotions in their children assessed by the Emotion-Related Parenting Styles (ERPS), (2) the parents’ emotion regulation capacity assessed by the Difficulties in Emotion Regulation Scale, short-form (DERS-SF), and (3) parents’ sense of self-efficacy and the strength of their relationship with the child will be assessed by the relationship with child scale (RWC) of the systemic inventory of change.

**Discussion:**

This study will provide insights into the effectiveness of EFST in improving children’s mental health and the mechanisms of change responsible for the program’s effectiveness. Impotently, this study may provide information regarding whether children’s mental health issues can be alleviated through therapeutic work provided to the parents alone.

**Trial registration:**

Clinical trials.gov Identifier: NCT04885036. First Posted on May 13, 2021. Trial status: In recruitment.

## Background

It is estimated that approximately 20% of children and adolescents experience some form of psychological distress that affects their level of functioning and quality of life and that approximately 7% are in need of professional assistance from specialist mental health care [1]. In Norway, mental health issues are estimated to cost society approximately 7 billion EURO per year [2]. Only around half of all children with mental health diagnoses will recover from their initial childhood mental health concerns by early adulthood [3]. Thus, effective, empirically supported, and cost-effective mental health programs are needed to address these issues more successfully. Today, a diversity of therapeutic approaches is offered in treating mental health difficulties among children and adolescents. Among these approaches are individual therapy [4], family therapy [5, 6], play therapy [7, 8], and group therapies [9]. While most of the approaches above have been shown to positively affect children’s and adolescents’ mental health status, many clients do not receive adequate treatment and care [10].

Parents play a key role in alleviating mental health problems in children and adolescents [11, 12]. Parental emotion awareness, emotion regulation, and response to their children’s emotions and their capacity to coach or guide children in how to recognize, understand, and regulate emotions, play a central role in the development of mental health in children [13, 14]. While the dominant approach in evidence-based parenting programs has been based on teaching behavioral strategies [5], recent findings support the importance of more emotion-focused interventions when parents and children experience emotion dysregulation [15]. Parental interventions are recognized as efficacious for the treatment of several mental health difficulties in children [5]. Even so, there is still a need to strengthen existing approaches and develop and test new interventions within this area. It is important to develop efficacious interventions when children are able and motivated to participate in therapy. However, it is also crucial to develop viable alternatives for situations when children do not wish to or are unable to participate in treatment. Emotion-Focused Skills Training (EFST) is an approach that aims to reach children and adolescents and alleviate mental health difficulties by *guiding parents to help their children recognize and cope with their emotions.* EFST was originally introduced as Emotion-Focused Family Therapy [16, 17] and has been further developed as a short-term parental skills training program (EFST). EFST is an integrative program based on principles from affective neuroscience, emotion theory, research on emotion-focused therapy [18], and humanistic person-centered therapy [19, 20].

EFST is an increasingly used treatment approach among therapists in outpatient clinics for child and adolescent mental health (i.e., BUP) in Norway. One example is the Department of Child and Adolescent Mental Health at Sorlandet Hospital (Abup) in Southern Norway, which has offered EFST to more than one hundred families during 2021. Prior studies examining the effects of EFST in community settings have demonstrated promising results, both when provided as a two-day caregiver workshop [21–23] and as a two-day workshop followed by individual sessions [24]. In a previous trial conducted by researchers affiliated with this current trial, two versions of EFST were compared in a randomized clinical dismantling study. Participants were parents of children with mental distress in a community sample. The standardized EFST protocol was compared to a psychoeducational version of EFST [24]. Results showed a significant decrease in both internalizing and externalizing symptoms, and the standard EFST protocol produced the most promising results. A weakness of the study was the absence of a comparison group other than the two EFST treatment variants. Thus, a comparison of EFST to a waitlist or active control group is necessary to determine its effectiveness.

Additionally, to our knowledge, the effect of EFST has not been thoroughly investigated in clients with more severe mental health problems and documented mental illnesses, such as those generally treated in Norwegian outpatient clinics for child and adolescent mental health. In a pilot study, members of our research team compared the effects of EFST to a group-based CBT treatment for children suffering from a clinical range of anxiety of different types (e.g., separation anxiety, social phobia, OCD). The results from the nine children included in this pilot study indicated that EFST had similar treatment effects as the CBT treatment (Zahl-Olsen R, Severinsen L, Stiegler JR, Shahar B, Bjerregaard Bertelsen T. Emotion Focused Skills Training for Parents with Anxious Children, a Pilot Study. 2022. Manuscript in preparation.). In the present study, we intend to compare the effectiveness of EFST intervention with treatment as usual (TAU). TAU in this study is the typical family-based intervention offered at the clinic (Department of Child and Adolescent Mental Health at Sorlandet Hospital, ABUP, in Southern Norway) which has already been documented as effective [25, 26]. There are reasons to anticipate that EFST effectiveness can be comparable to TAU.

EFST is based on the theory and practice of Emotion-focused therapy (EFT), which is a model for working with adult individuals and couples, and which has gathered extensive empirical support [27–30]. EFT is a process-oriented, humanistic-experiential, transdiagnostic approach based on the assumptions that emotional difficulties result from emotions that are no longer functional in helping the individual in an adaptive way [31–33]. EFT therapists support their clients in alleviating psychological distress by developing awareness of their emotions, labeling them into words, regulating their emotions, making meaning of their emotions, and transforming maladaptive emotions developed in the context of difficult experiences early in life [34]. The primary aim of EFST is to guide parents to become effective emotion coaches for their children. It is hypothesized that enhancing parents’ emotional awareness and ability to handle emotions will enable them to better understand and meet the child’s emotional needs, thereby improving the child’s emotional difficulties and reducing mental health difficulties [12]. However, to our knowledge, this assumption is not yet empirically tested.

## Aims and research questions

**Fig. 1:**
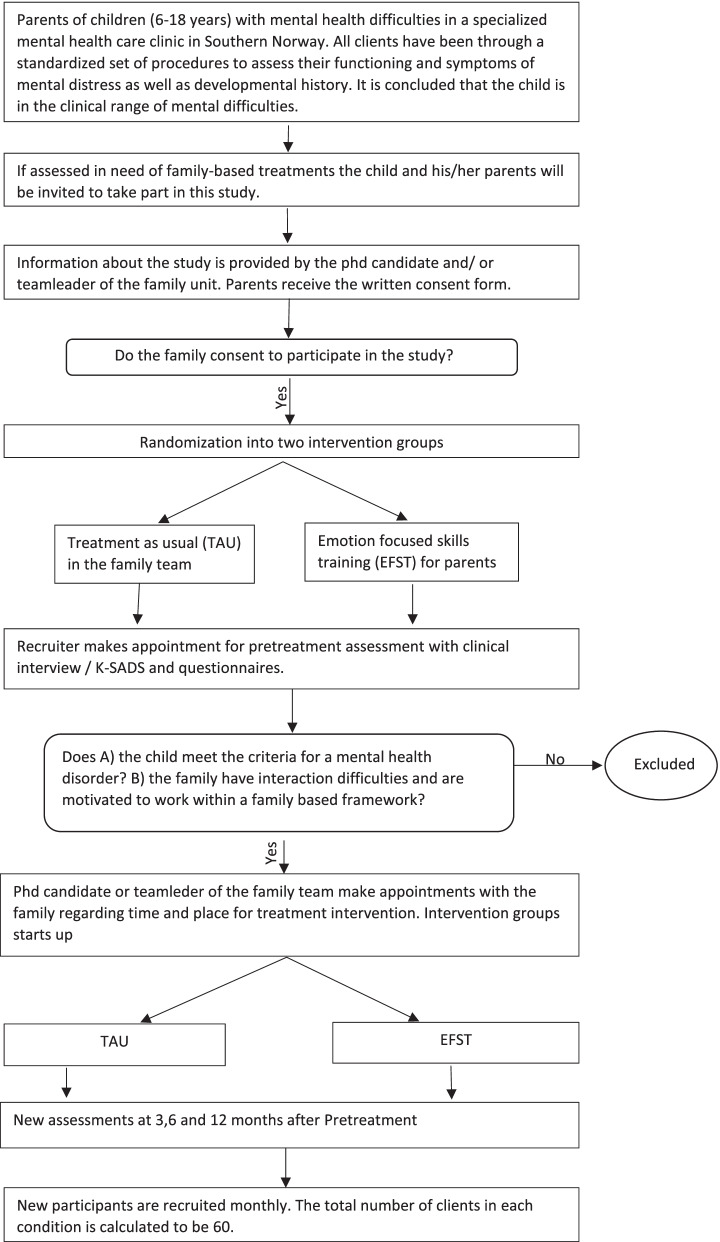
Flow of participants in the study

Using a randomized controlled trial in routine care, we aim to compare EFST with TAU within an outpatient clinic for child and adolescent mental health (BUP), as presented in Fig. 1. We have two main hypotheses. Our first hypothesis is that the two conditions are equivalent when it comes to effectiveness. Our second hypothesis is that an increase in parents’ emotional functioning predicts a decrease in mental health symptoms for their children.

Hence, the research questions we examine in this study are:

- 1. How does the EFST intervention compare to TAU in effectiveness?
- 2. Is there empirical evidence to support the EFST theory that parental increased emotional functioning can predict a decline in children’s mental health symptoms?

## Methods

### Study design

The proposed trial is a randomized controlled trial with two intervention groups. Participants are allocated into one of two groups, EFST or control group, TAU.

### Participants

Participants will be children referred to the child and adolescent mental health department at Sorlandet Hospital (Abup) in Southern Norway. As a standard procedure at the clinic, regardless of whether one takes part in a research project, all clients (i.e., children) undergo a standardized assessment of their functioning and symptoms of mental distress and developmental history. If, from the initial assessments, it is determined that the client is eligible for a family-based treatment in the family team of the clinic, the children and their parents will be invited to take part in this study. To be eligible, the following criteria must be met: a) the child meets the criteria for a mental health disorder, b) the disorder is assessed by health professionals to be related to parent or family functioning, and c) the families are motivated to work within a family-based framework to improve the suffering of their child and the relational difficulties in the family. The main researcher or the head of the family team will provide participants information about the study in age appropriate ways and go through the written consent form. After consent for participation, each family (one child with one or two parents) will be assigned randomly to one of the treatment conditions using the www.randomizer.org website. Randomization will be requested by the therapist responsible for recruitment and clinical interviews. Based on the clinic’s experience of research, an anticipated drop-out rate of 20% is reasonable [35, 36]. With 80% power, an expected effect size of 0.4, and an anticipated 20% drop-out rate, the number of clients in each condition is calculated to be 60, following a procedure described by Schönbrodt & Wagenmakers [37]. By choosing Bayesian statistics, evidence will be obtained even when the power calculations might be mis specified [38].

### Inclusion and exclusion criteria

As EFST is a transdiagnostic intervention, children with a wide range of clinical diagnoses (such as a variety of anxiety disorders and depression) as measured by K-SADS will be included. Children 6–18 years of age will be invited to participate in the project, and it is preferred that both parents participate in the EFST treatment (however, the family/or child can still be included if only one parent is able to participate). To be eligible for the study, parents must reside with their child and speak Norwegian well enough to understand and participate in sessions without an interpreter. Lastly, the child should not be receiving other psychotherapy at the clinic while in the trial.

### Retention

The main researcher of the trial will contact all parents included in the trial by telephone to ensure adequate information regarding the interventions, appointments and dates. Participants will also receive electronic links with reminders to respond to questionnaires.

### Research conditions

#### Condition 1: EFST Intervention

This condition consists of a parental EFST group-based workshop followed by 6 individual sessions. The workshop is conducted over two days, lasting a total of 13 h. Each group consists of 6–16 parents, ideally both parents of a referred child. In contrast to TAU, in this condition, the child does not participate in the treatment; only their parents do. The overall goal of EFST is to increase parents’ emotional competence and help parents work through their respective emotional barriers, such as fear and shame, that may prevent their effective parenting [12]. The manualized workshop utilizes psychoeducation experiential exercises and specific skills training. Two therapists conduct the workshop together. Individual supervision sessions will continue the process begun during the two-day workshop and will adhere to the EFST treatment manual [12]. EFST supports parents to follow their intention to engage actively in their child’s recovery process by increasing parental self-efficacy, emotional competence, and their ability to repair and strengthen the relationship with the child. Four core parenting skills are taught in the workshops and then elaborated and worked on in the individual sessions. The four core skills include:

- 1. *Validation of emotion*. Instead of trying to problem-solve or remove the child’s symptoms, parents are trained to recognize, understand, and communicate emotional understanding to their child. Being validated by parents when experiencing painful emotions has the potential to help children better understand and regulate their emotions [12].
- 2. *Enhancing motivation*. Parents are helped to understand and experience how their own problematic emotions, such as maladaptive shame and fear, can disrupt the parent–child interaction and prevent them from providing emotional guidance to their children.
- 3. *Resolving interpersonal injuries*. Parents are guided to take responsibility for ruptures in the relationship and repair past emotional injuries to strengthen the emotional bond with the child.
- 4. *Setting boundaries*. Building on the previous core skills, parents are helped to set healthy and flexible boundaries without judgment.

Guiding values for the therapist are the humanistic principles of empathy, unconditional positive regard, and genuineness [12, 39]. In the EFST individual sessions, evocative techniques (i.e., imaginative two-chair dialogues) are used as a central part of the intervention to help parents facilitate and engage in deeper emotional processing [12]. The parents will receive 19 h of EFST (13 h in group and 6 h of individual sessions). All EFST individual parent sessions will be videotaped to ensure adherence and provide data for a connected study that will further investigate the active mechanisms of change that may be at play in this intervention.

#### Condition 2: TAU

In the TAU condition, families referred to the outpatient mental health clinic receive the most commonly offered family-based treatment at the clinic. This is a non-manualized systemic integrative family therapy where treatment length varies, but with an observed mean of 14 sessions based on data at the clinic. The exact type of intervention is established in collaboration with the family. In contrast to more individual-based therapies, the child participates in the therapy as well as parents and other family members. The family and the relationships between family members are viewed as critical resources for treating mental health issues [40]. In TAU, there are always two therapists working with each family. The therapists are a part of an interdisciplinary team of clinicians that discuss and receive feedback regarding the therapeutic processes. Recent research conducted by members of our research group has shown that this type of TAU is effective [25, 26]. To obtain a more precise and detailed description of the TAU offered in this trial, we will collect information about the intensity and type of therapy provided by therapists in conjunction with more objective measurements obtained from the medical record/journal.

### Therapists

Therapists in the TAU condition are all qualified mental health professionals employed at the clinic’s family team. The team (*N* = 12) consists of certified family therapists, psychologists, and one psychiatrist.

Five qualified EFST therapists employed on the family team (three family therapists and two psychologists) will conduct the EFST intervention. During the data collection, the EFST therapists will gather monthly for three-hour supervision sessions to discuss video material, dilemmas, technical and ethical considerations, and ensure treatment fidelity.

### Measurements

To investigate the research questions, a combination of evaluations and scores from professionals administering the Schedule for Affective Disorders and Schizophrenia for School-Age Children–Present and Lifetime version (K-SADS PL; [41]) and self-evaluation questionnaires with solid psychometric properties will be used at the time points presented in Table 1. As shown, T0 is at pretreatment, T1 after three months, T2 after six months, and T3 after 12 months. With TAU not being a manualized intervention with a fixed length of treatment, we decided to measure the symptoms of the child 3 months after starting the program. At that time, the EFST treatment is terminated, and TAU has reached the point in treatment (if not ended) where we have reason to believe that the treatment has had an effect.

**Table 1:** Measures and timeline of assessment

**Measures**	**T0**	**T1**	**T2**	**T3**
K-SADS	x	x		
SDQ	x	x	x	x
KINDL-R	x	x	x	x
DERS-SF	x	x	x	x
ERPS	x	x	x	x
WAI		x		
SCL-10	x	x		
RWC	x	x		

#### Measures for research question 1

To answer our first research question, *How does the EFST intervention compare to TAU in effectiveness*, we will use both a semi-structured interview and parent, teacher, and self-report measures. Our primary outcome measure is the Schedule for Affective Disorders and Schizophrenia in School-Age Children-Present and Lifetime Version (K-SADS-PL), a semi-structured diagnostic interview conducted by qualified clinicians. The interview will be conducted at T0 and T1 to investigate the presence of one or more mental health diagnoses of the children. A diagnosis will be rated as present or not as described in the interview guide [42]. K-SADS-PL is a semi-structured diagnostic interview used to evaluate current and lifetime episodes of psychopathology in children and adolescents. The measure can be used to evaluate at least 33 psychiatric disorders in children and adolescents [43]. In the proposed study, the K-SADS-PL interview will be conducted with the child and parent together. International studies suggest good validity and reliability of the K-SADS-PL [41, 44, 45], and it is validated for the Norwegian population [43].

Our second outcome measure, the Strength and Difficulties Questionnaire (SDQ) [46], is a 25-item questionnaire completed by parents, children (from 11 years of age), and teachers to assess mental health, peer relations, and prosocial behavior. SDQ will be measured at all time points and takes only a few minutes to complete. International and Norwegian studies found satisfactory reliability and validity, with best results for parent reports and generally lowest performance for self-reports [47–49].

In addition to measuring symptoms and problem areas, we will investigate the children’s perceived quality of life at all time points answered by the parents and children. For this purpose, the 24-item self-report measure Health-Related Quality of Life (KINDL-R) [50] will be used. International and Norwegian studies found satisfactory to good validity and reliability of the measure [51, 52].

#### Measures for research question 2

To answer the second research question, *is there empirical evidence to support the EFST theory that parental increased emotional functioning can predict a decline in children’s mental health symptoms,* we apply three perspectives.

The first perspective, how parents relate to their children's emotions, will be assessed by the 20-item self-report measure Emotion-Related Parenting Styles (ERPS) [53] at all time points. International studies have confirmed the reliability and validity of the measure [54, 55]. Our second perspective, the parents’ emotion regulation, will be assessed by the 18-item self-report measure Difficulties in Emotion Regulation Scale, short-form (DERS-SF) [56] at all time points. International studies have confirmed the reliability and validity of the measure [57].

Our final perspective, parents’ sense of self-efficacy and the strength of their relationship with the child, will be assessed by the relationship with child scale (RWC) of the systemic inventory of change [58] at all time points. International studies have confirmed the reliability and validity of the measure [59, 60].

#### Additional measures

To assess the parents’ mental health, the Hopkins Symptom Checklist-10 (SCL-10) will be used at T0 and T1. SCL-10 is a short form of the longer SCL-90. A large Norwegian study showed that the shorter versions of SCL performed almost as well as the full version [61], and international research has confirmed the reliability and validity of the measure [62, 63].

Due to the fact that alliance is a significant predictor of outcome across ages and treatments [64], it is considered important to determine whether the alliances in the two intervention groups are comparable. To control for alliance in the two conditions, the Working Alliance Inventory (WAI) [65] will be assessed by parents at T1. WAI has been found to have adequate validity and reliability [65].

### Data analyses

To test our first hypothesis that the two conditions are equivalent when it comes to effectiveness, Bayesian growth curve analysis and t-tests will be used to assess the effect of the two arms as compared to each other. Independent sample t-test will be performed on the SDQ to compare the outcome of the two conditions for every five clients included in the study. If strong evidence of difference of the treatment effect is provided with few clients, the data collection can stop. To test our second hypothesis that an increase in parents’ emotional functioning predicts a decrease in mental health symptoms for their children, Bayesian growth curve analyses will be used. Bayesian analysis is preferred because it allows for the specification of models that are appropriate for the data and the capacity to provide estimates and credible intervals for any derived parameter. Additionally, when using Bayesian analysis, credible intervals are not dependent on large-N approximations (as confidence intervals used in frequentist statistical approaches), nor are credible intervals dependent on the type of test being performed (as confidence intervals do in frequentist approaches). Furthermore, Bayesian analysis gives methods for evaluating support for the null hypothesis, not just against it [66]. Moreover, it enables monitoring of data collection to see whether there is sufficient evidence to draw conclusions or if additional data is required [67]. Indeed, there is evidence that within child and adolescent mental health research, conclusions could have been made with smaller sample sizes than what was obtained in many cases [68]. The effect size of interest is the difference in treatment effect between each of the arms in the study. Effect sizes of treatments within our field vary [10, 69], with treatment effects of 0.80 regarded as good. In line with conventional procedures in meta-analyses [65], we consider a difference of 0.40 as meaningful. Based on the experience of research within the clinic, an anticipated drop-out rate of 20% is reasonable [35, 36]. Clinical significant change will be assessed using the method defined by Jacobson & Truax [54]. Missing data will be managed with multiple imputation and all analyses will be performed as intention to treat (ITT).

### Ethical considerations

The procedure, study design and informed consent form were approved by the Regional Committee for Medical and Health Research Ethics in Norway (REK no. 229366) as well as approval by the hospitals own research department. Changes in the protocol or procedure will be reported to the committee. All participants are evaluated at the child and adolescent mental health department. Mental health professionals are involved in both intervention groups. If it is determined that the child, parents or family requires additional interventions, this will be addressed. All participants will be informed that they can withdraw their consent at any time and still receive the mental health interventions needed.

### Data management

An elaborate data management plan has been approved by the hospital’s research department. All data will be stored according to the rules and regulations of the hospital to ensure confidentiality of the data at all stages of the research process. All study-related information will be stored securely at the study site. All participant information will be stored in locked file cabinets in areas with limited access. Only the main researchers and one research assistant will have access to the data.

### Dissemination policy

Study results will be disseminated to the public in peer-reviewed journals and at academic conferences. The order of presenters and authors will be decided based on the contributions of each member.

## Discussion

With EFST being a newly developed method, there is still a lack of empirical evidence on the effectiveness of this method and a need for RCT studies. The present study will provide important contributions to this field. Despite limited evidence of EFST's effectiveness, it is increasingly being used in outpatient clinics in Norway for child and adolescent mental health. To assess whether the intervention should continue to be offered, it is necessary to conduct an evaluation of its effectiveness. This project will supplement previous research to facilitate evidence-based decision-making in mental health clinics. If it is proved to be as beneficial as TAU, it should be supplied to a greater number of families than is currently the case since it is considered to be an easy to implement and cost-effective treatment with minimal burden to the child. In contrast, if the intervention is determined to be unsuccessful, clinics must consider how it should be used and to whom it should be offered. Additionally, this study will provide insight that may be used to question and broaden the EFST theory regarding the relationship between changes in a parent's emotional process and changes in a child’s mental health symptoms.

Additionally, from a study design standpoint, experiences with data monitoring during data collection can serve as a reference for future research in the field of child and adolescent mental health.

### Timeline for completion of the study

Data collection starts in December 2021 and is estimated to be finished in June 2023.

## Data Availability

Data sharing is not applicable to this article as no datasets were generated or analyzed in this study protocol. However, we will grant public access to anonymized participant level data and statistical code used in research articles published from this study. The corresponding author can be contacted to obtain the anonymized data.

## Abbreviations

ABUP: The Department of child and adolescent mental health at Sorlandet Hospital
ADIS-IV-C/P: Anxiety disorders interview schedule for child/parent interview schedule
BUP: Child and adolescent psychiatric outpatient clinic
DERS: Difficulties in Emotion Regulation Scale
EFT: Emotion-Focused Therapy
EFST: Emotion-Focused Skills Training
ERPS: Emotion-Related Parenting Styles
KINDL-R: Health-Related Quality of Life scale
K-SADS: Schedule for Affective Disorders and Schizophrenia, present and lifetime version
OCD: Obsessive–compulsive disorder
RCT: Randomized controlled trial
RCI: Reliable Change Index
REK: The Regional Committee for Medical and Health Research Ethics in Norway
RWC: Relationship with child scale
SDQ: Strength and Difficulties Questionnaire
TAU: Treatment as usual
WAI: Working Alliance Inventory
